# The Value of PETRA in Pulmonary Nodules of <3 cm Among Patients With Lung Cancer

**DOI:** 10.3389/fonc.2021.649625

**Published:** 2021-05-18

**Authors:** Hui Feng, Gaofeng Shi, Hui Liu, Yu Du, Ning Zhang, Yaning Wang

**Affiliations:** Department of Radiology, The Fourth Hospital of Hebei Medical University, Shijiazhuang, China

**Keywords:** lung nodule, MRI, CT, PETRA, malignancy

## Abstract

**Objective:**

This study aimed to evaluate the visibility of different subgroups of lung nodules of <3 cm using the pointwise encoding time reduction with radial acquisition (PETRA) sequence on 3T magnetic resonance imaging (MRI) in comparison with that obtained using low-dose computed tomography (LDCT).

**Methods:**

The appropriate detection rate was calculated for each of the different subgroups of lung nodules of <3 cm. The mean diameter of each detected nodule was determined. The detection rates and diameters of the lung nodules detected by MRI with the PETRA sequence were compared with those detected by computed tomography (CT). The sensitivity of detection for the different subgroups of pulmonary nodules was determined based on the location, size, type of nodules and morphologic characteristics. Agreement of nodule characteristics between CT and MRI were assessed by intraclass correlation coefficient (ICC) and Kappa test.

**Results:**

The CT scans detected 256 lung nodules, comprising 99 solid nodules (SNs) and 157 subsolid nodules with a mean nodule diameter of 8.3 mm. For the SNs, the MRI detected 30/47 nodules of <6 mm in diameter and 52/52 nodules of ≥6 mm in diameter. For the subsolid nodules, the MRI detected 30/51 nodules of <6 mm in diameter and 102/106 nodules of ≥6 mm in diameter. The PETRA sequence returned a high detection rate (84%). The detection rates of SN, ground glass nodules, and PSN were 82%, 72%, and 94%, respectively. For nodules with a diameter of >6 mm, the sensitivity of the PETRA sequence reached 97%, with a higher rate for nodules located in the upper lung fields than those in the middle and lower lung fields. Strong agreement was found between the CT and PETRA results (correlation coefficients = 0.97).

**Conclusion:**

The PETRA technique had high sensitivity for different type of nodule detection and enabled accurate assessment of their diameter and morphologic characteristics. It may be an effective alternative to CT as a tool for screening and follow up pulmonary nodules.

## Introduction

Pulmonary nodules are common findings in individuals with an elevated risk for lung cancer. According to the Fleischner Society, the risk of malignancy in nodules of <6 mm in diameter is extremely low, while nodules of >6 mm in diameter require multiple follow-ups or further evaluation (1, 2). The National Lung Screening Trial demonstrated the benefits of using multi-slice computed tomography (CT) for lung cancer screening in a high-risk population. This approach was shown to reduce lung cancer-associated mortality by up to 20% (3, 4). Results of NELSON trial showed that lung-cancer mortality was significantly lower among those who underwent volume CT screening than among those who underwent no screening (5). Although this technique has high spatial resolution and good contrast between air and lung tissues, repetitive use of CT increases the risk of developing cancer due to ionizing radiation exposure, particularly in patients who require reviews on multiple occasions over a prolonged period (6, 7).

Magnetic resonance imaging (MRI) is a non-ionizing technique, which is of particular interest for the assessment of lung diseases, including pneumonia and cystic fibrosis, in children or patients who require frequent follow-up examinations (8–11). Continuous technical developments in terms of stronger gradients, parallel imaging, and shorter echo times have led to increased interest in MRI as an alternative to CT for lung screening (12, 13).

Short echo times have been shown to be crucial in improving the quality of lung proton MRIs (14). The pointwise encoding time reduction with radial acquisition (PETRA) sequence is a noiseless prototype hybrid approach to ultrashort-echo-time three-dimensional imaging (15, 16). This technique offers several advantages over other conventional sequences, including three-dimensional isotropic imaging with submillimeter spatial resolution (17). Therefore, the present study was conducted to evaluate the detection and sizing of different subgroups of lung nodules using the PETRA sequence with a 3T MRI scanner and compare these results with CT findings.

## Materials and Methods

The present prospective study was approved by the institutional review board and the federal agency for radiation protection. Written informed consent was obtained from each participant.

### Patient Population

Between May 2017 and September 2018, patients from a lung cancer screening program, were enrolled for the present prospective study according to the recommendations of the Fleischner Society’s guidelines regarding relevant risk factors (18). Inclusion criteria: 1) patients diagnosed with lung cancer, and nodule<3cm in diameter; 2) patients with a time interval of <24 h between their CT scan and MRI; and 3) patients >18 years old. Exclusion criteria: 1) patients who had pacemakers; 2) patients who had metal implants; 3) patients with severe claustrophobia; and 4) patients suffering from severe emphysema and/or fibrosis.

### CT Imaging Protocol

For acquisition, the patients were in the supine position on a 128-row multi-detector dual source CT (Definition Flash, Siemens Healthcare, Forchheim, Germany). The CT protocol used included the following parameters: tube voltage = 120 kV; 50 mAs; pitch = 1.2; field of view (FOV) = 300 mm; slice thickness = 1 mm; and kernel = B70f. The patients were asked to raise their arms and hold their breath, so the scanning range included the area from the chest entrance to the diaphragm, thus covering the whole lung. The scan time was 3.98 s, and the effective dose was 1.2–1.5 mSv.

### CT Image Analysis

The CT images were evaluated in clinical routine by a radiologist with ten years of experience (N.Z.), who had no knowledge of the MRI findings. The location, size, and type of each lung nodule were recorded. In line with the literature (19–21), the nodules were classified into three groups according to density: solid nodules (SNs), partial solid nodules (PSNs), and ground glass nodules (GGNs).

Following the recommendations of the Fleischner Society (19), the nodules were categorized based on size: SNs of <6 mm, 6–8 mm, or >8 mm in diameter; and PSNs of <6 mm or ≥6 mm in diameter. Nodule size was defined as the mean value of the longest and shortest axial diameters. Furthermore, the nodules were categorized according to location as follows: left lung (upper and lower lobe groups); right lung (upper lobe group, middle lobe group, and lower lobe group); center group (distance from the peripheral pleura of >1 cm); and peripheral group (distance from the peripheral pleura of <1 cm). The largest level of each nodule was selected for measurement. All nodules were measured in the standard lung window setting (window center = −600 HU; window width = 1,200 HU).

The CT was considered the gold standard for recording the number of detected nodules. Thus, a false positive result was defined as a nodule diagnosed by MRI that was not observed in the CT images, and a false negative result was defined as a nodule diagnosed in the CT images that was not observed by MRI.

### MRI Protocol

MRI was performed using a 3T MRI unit (Magnetom Skyra, Siemens Healthcare). A multi-channel phased-array receiver body coil was used for detection. The patients assumed the supine position, with their arms raised above their body, and they were instructed to breathe freely. The PETRA sequence parameters were as follows: TR = 3 ms; TE = 0.07 ms; matrix = 256 × 256; FOV = 400 × 400; slice thickness = 1.6 mm; and acquisition plane = axial. To reduce cardiac and breathing motion artifacts, respiratory gating was performed. The total scan time was 3 min.

### MRI Analysis

The prospective MRI data analysis was performed by two radiologists with ten years (N.W., observer 1) and five years (Y.D., observer 2) of experience, respectively. All MRI datasets obtained from the 75 participants were pseudonymized and presented in random order. To eliminate detection bias, both of these observers were unaware of the CT findings. The recorded content for each nodule included the location of the nodule (including the lung lobe and segment), the position in the lung parenchyma (center or peripheral), and size. Morphologic characteristics in CT and MRI were assessed by both readers for each nodules. The characteristics including shape (round, oval or irregular), margins (smooth, nonsmooth, lobulated, spiculated, pleural tag) and attenuation (GGN, SN or PSN). Any disagreements between the radiologists’ findings were resolved by consensus interpretation.

To investigate intraobserver variation, observer 1 measured the nodules twice after one month. We used the average value of measurements the final reference value of MRI measurement to compare with CT.

### Statistical Analysis

The statistical analysis was performed using SPSS 21 (IBM, Armonk, NY, USA). The sensitivity levels of detection for the different subgroups of pulmonary nodules were calculated according to location, size (based on the Fleischner Society’s nodule size categorization guide), and type. The measures of diagnostic performance were calculated for the nodule detection by MRI, and these results were then compared with the CT findings (the gold standard of nodule detection). Comparison of categorical variables was performed using Chi-square test and McNemar’s test. Morphologic features from CT and PETRA were compared using Kappa test, with the following levels of agreement (22): 0-0.20,poor; 0.21-0.40, fair; 0.41-0.60, moderate; 0.61-0.80, substantial; and 0.81-1.00, almost perfect agreement. The mean nodule diameters from the CT and PETRA findings were compared using Bland–Altman plots. Interobserver and intraobserver agreement rates were assessed by intraclass correlation coefficient (ICC). Receiver operating characteristic(ROC) analyses were performed to determine feasible threshold values for the mean diameter of detected nodules and to evaluate the usefulness of PETRA sequence. An error probability of P < 0.05 was considered to be statistically significant.

## Results

### General Characteristics

According to the recommendations of the Fleischner Society regarding relevant factors, a total of 82 patients from a lung cancer screening program (36 males and 46 females; mean age = 58; age range = 52–85) were enrolled for this prospective study. The exclusion criteria included patients who had contraindications to MRI, including pacemakers, metal implants, and severe claustrophobia, and patients with severe emphysema and/or fibrosis. Based on the inclusion and exclusion criteria, 75 patients who underwent low-dose CT (LDCT) and MRI (31 males and 44 females; mean age = 56.5; age range = 50–75) were ultimately included in the study.

### Lung Nodule Detection Rates

The CT imaging revealed 256 lung nodules (mean diameter: 8.3 ± 4.6 mm) among the 75 sample patients, comprising 99 SNs, and 157 subsolid nodules (including 86 PSNs and 71 GGNs). Among the SNs, 47 nodules measured <6 mm in diameter, 20 nodules measured between 6 and 8 mm in diameter, and 32 nodules measured >8 mm in diameter. Among 157 subsolid nodules, 51 nodules measured <6 mm in diameter, and 106 nodules were >6 mm in diameter. The mean number of nodules per patient was 3, with a range of 1–20. From CT examination, the diameter of the nodules ranged from 2.4 to 24.3 mm. A breakdown of the patient characteristics is presented in **Table 1**.

**Table 1:** Patient characteristics.

Characteristics	Finding
Age(y)	56.5±10.5 (50-75)
Male(n=31)	56.2±12.5 (52-75)
Female(n=44)	56.7±9.44 (55-70)
Nodule location	
Middle and lower lobe	127
Upper lobe	129
Nodule type	
SN	99
PSN	86
GGN	71
Diameter(mm)	
Overall	8.3±4.6 (2.4-24.3)
SN	6.9±3.4 (2.4-23.1)
PSN	12.5±4.5 (5.1-24.3)
GGN	7.6±3.1 (3.8-14.8)

**Figure 1:**
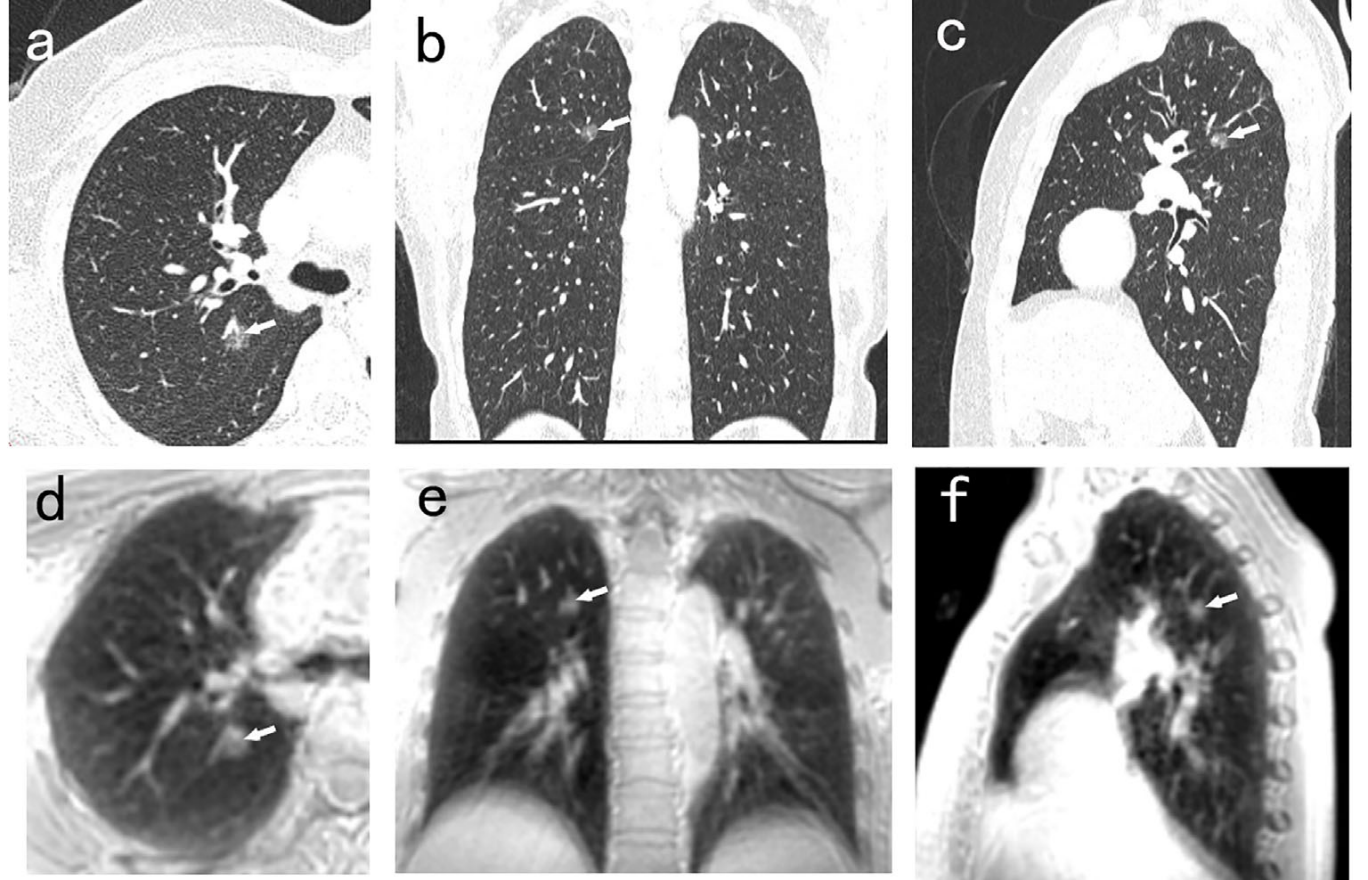
A 50-year-old man with 8.8mm GGN (arrow) in the right upper lobe. The nodule is well depicted on the CT axial **(A)**, coronal **(B)**, and sagittal **(C)** image, and on the PETRA sequence axial **(D)**, coronal **(E)**, and sagittal **(F)** image. The arrows represent the locations of nodules.

**Figure 2:**
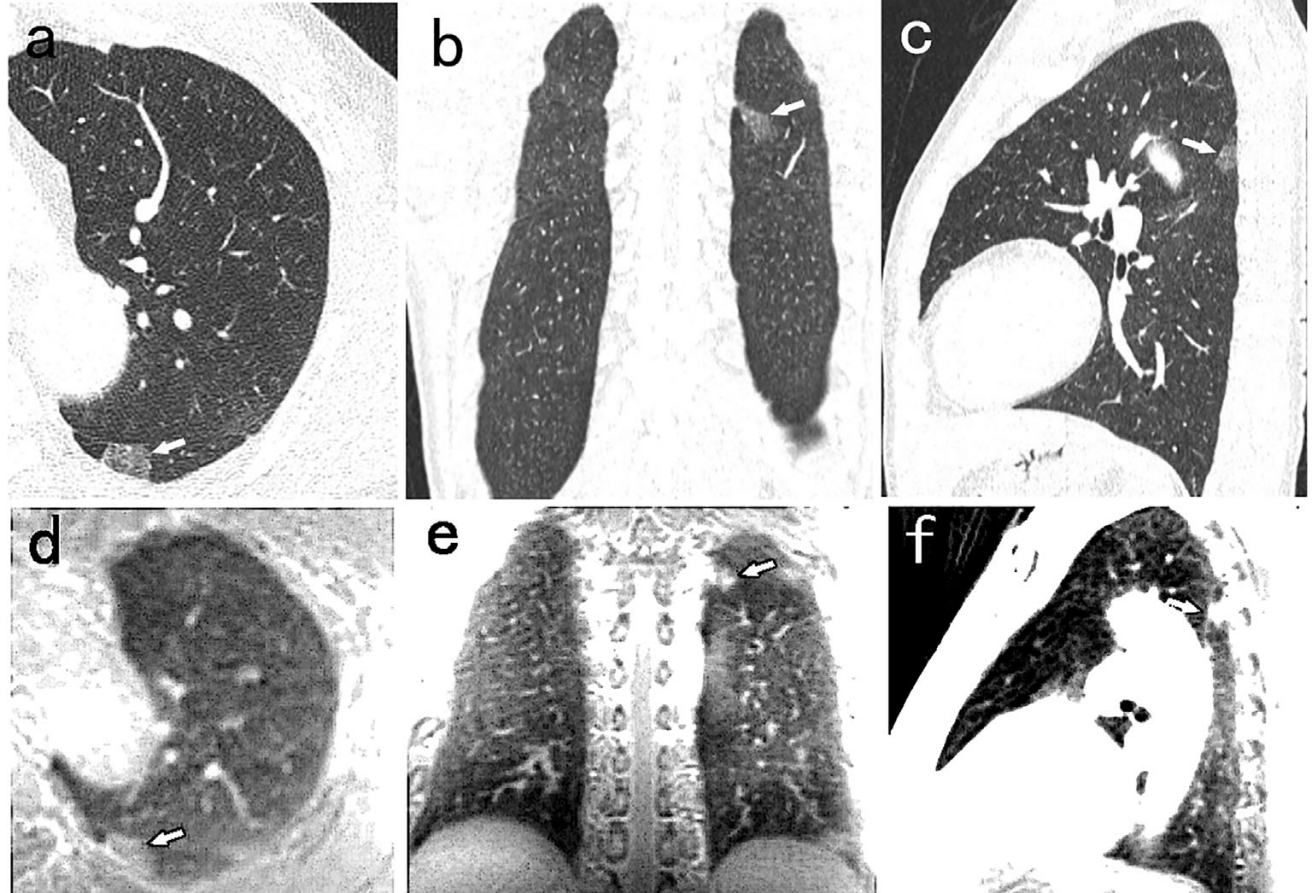
A 52-year-old man with 14mm PSN (arrow) in the left lower lobe. The nodule is well visualized on the CT axial **(A)**, coronal **(B)**, and sagittal **(C)** image, and on the PETRA sequence axial **(D)**, coronal **(E)**, and sagittal **(F)** image. The arrows represent the locations of nodules.

**Figure 3:**
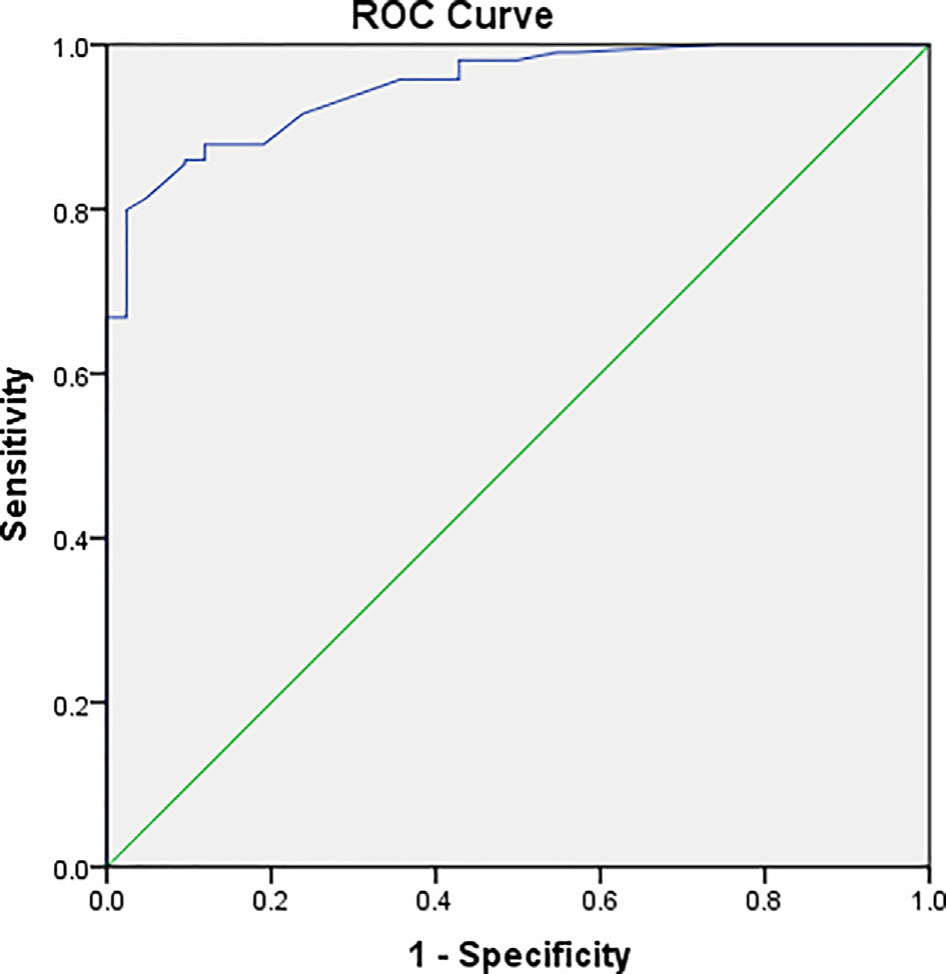
Graph illustrating the result of ROC analyses. ROC analysis was performed according to the mean diameter of the nodules. ROC curve showed the diagnostic perfomance of PETRA sequence in detecting nodules, AUC=0.946. Nodules with a diameter greater than 6mm, the sensitivity and specificity of detecting are 85.5% and 99.5%.

The PETRA sequence accurately detected 214 of 256 pulmonary nodules, but it failed to identify 42 nodules, and the overall sensitivity was 84%. For pulmonary nodules >8 mm in diameter, the detection rate was 100% (**Table 2**). MRI had a higher detection rate for SNs (82%) and PSNs (94%) than for GGNs (72%) (**Figures 1** and **2**). ROC curve (**Figure 3**). Showed the diagnostic perfomance of PETRA sequence in detecting nodules, AUC=0.946. Nodules with a diameter greater than 6mm, the sensitivity and specificity of detecting are 85.5% and 99.5%.

**Table 2:** The sensitivity of the detection of different subgroups of pulmonary nodules (according to the Fleischner Society nodule size category) and different lobe.

Diameter of pulmonary nodules	Number of nodules detected on CT	Number of noudles detected on MRI	p value	Sensitivity of MRI	False positive
Solid nodule	99	82	0.143	82%	2
<6mm	47	30		64%	2
6-8mm	20	20		100%	0
>8mm	32	32		100%	0
Subsolid nodule					
PSN	86	81	0.566	94%	0
<6mm	19	15		79%	0
≥6mm	67	66		99%	0
GGN	71	51	0.080	72%	7
<6mm	32	15		47%	4
≥6mm	39	36		92%	3
Total	256	214		84%	9

Data are number of noudles detected by CT and MRI.

P value was analyzed by using chi-square test, indicating that there was no statistically significant difference in the detection rate of pulmonary nodules of different sizes in MRI.

### The PETRA Had a Higher Sensitivity of for Nodules Located in the Peripheral Lobe

Compared with nodules located in the middle or lower lobes, the PETRA sequence had a higher detection ability in the upper lobe, and nodules located in the peripheral lobe were more easily detected. The undetected nodules were predominantly located close to the diaphragm or around the heart (**Table 3**).

**Table 3:** No. of Lesions Detected on CT and MRI (Periphery and Center).

Lobe	Number of nodules	Detected by MRI	p value	Sensitivity of MRI
Periphery				
Upper	62	56	0.125	90%
Middle and Lower	70	59	0.118	84%
Total	132	115	0.109	87%
Center				
Upper	67	60	0.070	89%
Middle and Lower	57	39	0.011	68%
Total	124	99	0.001	80%

Data are number of noudles.

P value was analyzed by McNemar method, and there was no statistical difference between the two methods of CT and MRI in the group of upper lobe and middle and lower lobe of peripheral nodules. In the central region, the detection rate of the upper lobe group was higher than that of the middle and lower lobe group, and the difference was statistically significant.

The range of nodule diameters detected by MRI was 2.9–24.3 mm, and the mean diameter was 8.6 mm. The maximum diameter of the largest lesion not detected by MRI was 7.3 mm. For nodules >6 mm in diameter, the PETRA sequence achieved 97% sensitivity. All of the pulmonary nodules falsely reported by MRI had diameters of <6 mm.

**Figure 4:**
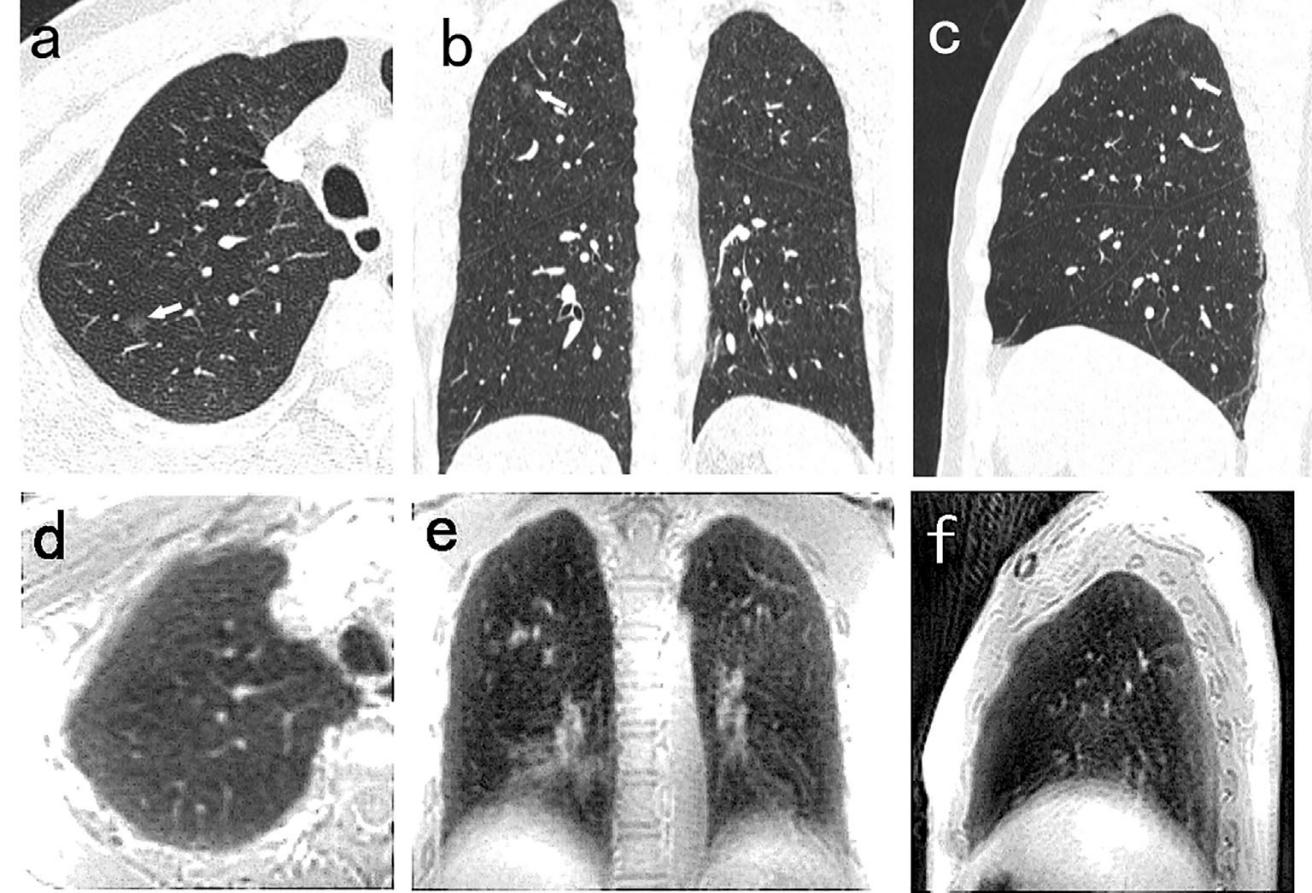
A 54-year-old man with 5.5mm GGN (arrow) in the right upper lobe. The nodule is well visualized on the CT axial **(A)**, coronal **(B)**, and sagittal **(C)** image, and is not detected in the PETRA sequence **(D–F)**. The arrows represent the locations of nodules.

There were nine false positive nodules identified by the PETRA sequence. The diameters of six of the false positive nodules were <6 mm, and five of the nine nodules were close to the diaphragm or around the heart. The sizes of the nodules undetected by MRI were as follows: 38 nodules of <6 mm diameter, and eight nodules of ≥6 mm diameter. Among these 42 undetected nodules, SNs, PSNs, and GGNs accounted for 40% (17/42), 12% (5/42), and 48% (20/42), respectively (**Figure 4**).

### Strong Agreement Between CT and PETRA

**Figure 5:**
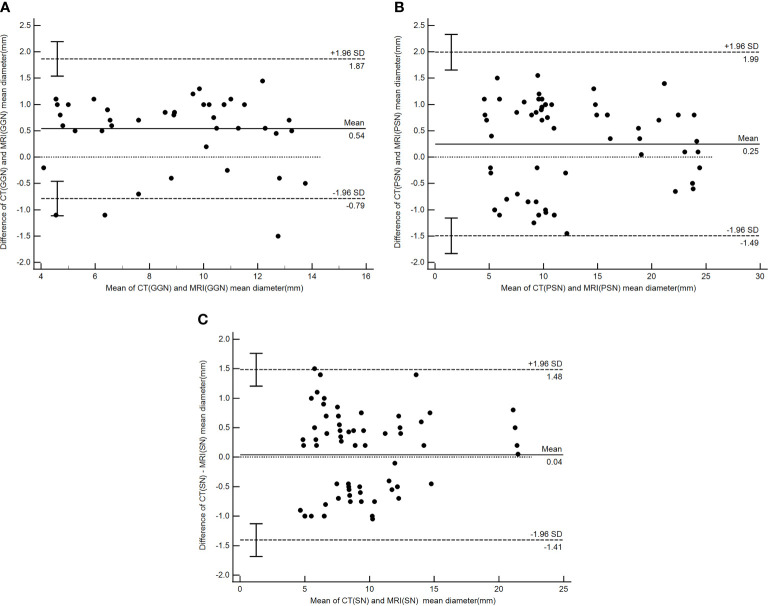
Bland–Altman plot showing the agreement between the nodule mean diameter measurements by CT and PETRA for GGN **(A)**, PSN **(B)** and **(C)** SN respectively. Dashed lines denote 95%CI, and solid lines denote mean.

There were good levels of both interobserver and intraobserver agreement regarding the mean diameters of pulmonary nodules measured by CT and MRI, and the ICC results were 0.990 and 0.992, respectively (**Table 4**). The Bland–Altman plots present the differences between mean diameters from the CT and MRI results for GGNs, PSNs and SNs respectively (**Figure 5**). The results of agreement between CT and MRI to assess the morphologic characteristics of detected nodules was found to be substantial or almost perfect. (K=0.816-1.00) (**Table 5**).

**Table 4:** Mean diameter of detected nodules for subgroups and agreement of measurements compared to CT.

Items	CT	PETRA				
		Observer1	Observer1*	ICCintra	Observer2	ICCinter
Solid nodule						
<6mm	4.94±0.59*	4.43±0.64*	4.34±0.62*	0.963[0.933;0.979]^¤^	4.54±0.53*	0.954[0.916;0.975]^¤^
6-8mm	7.77±1.15*	6.84±1.33*	7.05±1.30*	0.980[0.963;0.990]^¤^	7.15±1.04*	0.970[0.943;0.985]^¤^
>8mm	11.66±2.49*	11.45±2.57*	11.46±2.51*	0.996[0.993;0.998]^¤^	11.36±2.54*	0.990[0.982;0.985]^¤^
PSN						
<6mm	5.10±0.55*	4.56±0.57*	4.44±0.50*	0.961[0.937;0.976]^¤^	4.54±0.59*	0.966[0.945;0.979]^¤^
≥6mm	16.31±4.85*	14.83±4.81*	15.18±4.75*	0.995[0.991;0.997]^¤^	15.16±4.64*	0.996[0.994;0.998]^¤^
GGN						
<6mm	5.07±0.56*	4.41±0.61*	4.29±0.67*	0.938[0.901;0.962]^¤^	4.50±0.59*	0.898[0.836;0.937]^¤^
≥6mm	9.90±2.47*	8.89±2.52*	8.76±2.27*	0.990[0.948;0.994]^¤^	9.10±2.19*	0.987[0.979;0.992]^¤^
Total	8.30±4.60*	7.53±3.24*	7.48±2.96*	0.992[0.976;0.995]^¤^	7.43±2.85*	0.990[0.977;0.994]^¤^

*Data are mean±standard deviation. ^¤^Data are ICC value [minimum;maximum].

Observer1* measurement second time by observer1.

ICCintra Intraobserver reproducibility assessed by intraclass correlation coefficient.

ICCinter Interobserver reproducibility assessed by intraclass correlation coefficient.

**Table 5:** Comparison and agreement between CT and MRI to assess the morphologic characteristics of detected nodules.

Nodule Characteristic	CT	PETRA	Kappa value
Shape			0.957
Round or oval	147(68.6%)	145(67.7%)	
irregular	67(31.4%)	69(32.3%)	
Margins			0.946
smooth	149(69.6%)	144(67.2%)	
nonsmooth	65(30.4%)	70(32.8%)	
Lobulated	46(21.4%)	40(18.6%)	0.913
Spiculated	15(7%)	12(5.6%)	0.882
Pleural tag	10(5%)	7(3.2%)	0.816
Attenuation			
Solid nodule	82(38.3%)	82(38.3%)	1.00
Partial solid nodules	81(37.8%)	80(37.3%)	0.98
Ground-glass nodule	51(23.9%)	48(22.4%)	0.95

Data are number(%) of nodules.

## Discussion

The value of MRI in patients with pulmonary nodules in terms of both lesion detection and characterization has been assessed in several studies (2, 23, 24). The reported sensitivity of nodule detection using MRI ranges between 40% and 93% (2, 15–21, 23–34). The PETRA sequence is a noiseless prototype hybrid approach to ultra-short-echo-time three-dimensional imaging (15, 18) that does not require a hardware change. The rapid imaging with ultra-short echo time enabled by PETRA may be useful for new clinical applications (17). Dournes et al. (17) demonstrated that the PETRA sequence offers advantages for lung imaging, including noiseless, free-breathing, and isotropic imaging. Ohno et al. (35) assessed the nodule detection capability of standard-dose CT, reduced-dose CT, and MRI with ultrashort echo time (UTE), and the sensitivity of the UTE technique was >90%. In the present study, the capability of the PETRA sequence in nodule detection was as effective as CT, and the PETRA sequence achieved high sensitivity (84%) for nodule detection. The sensitivity of MRI for the detection of pulmonary nodules with diameters of >6 mm was 97% (152/158), and for nodules with diameters of >8 mm, the sensitivity was 100%. For nodules with diameters of >6 mm and >8 mm, the detection rate reached 97% and 100%, respectively. The results of the present study are compatible with those presented by Ohno et al. (35). Furthermore, the present study indicated the superiority of the PETRA sequence in detecting GGNs (diameter of ≥6 mm; detection rate of 97%), which is also similar to the results reported by Ohno et al. (35). The present study demonstrated that PETRA can be an optimal sequence for the detection of lung lesions. This is consistent with the work of Dournes et al. (17), who highlighted the superior performance of PETRA compared with the T1-VIBE sequence (36). ROC curve showed the diagnostic perfomance of PETRA sequence in detecting nodules, AUC=0.946. Nodules with a diameter greater than 6mm, the sensitivity and specificity of detecting are 85.5% and 99.5%. According to the recommendations of the Fleischner Society guidelines, only nodules of ≥6 mm in diameter and growing nodules are associated with a risk of malignancy (Lung-RADS category 3 and 4) (37). Therefore, the results of the present study fit well with the sensitivity of MRI for nodule detection as reported in previous studies (30, 32, 38, 39).

In the present study, nine false positive nodules were identified by the PETRA sequence, giving a false positive rate of 3.5%. In other studies, false positive rates have ranged between 5% and 8.7% (28, 36). False positives have frequently been found in the T2-TSE sequence rather than in the T1-VIBE sequence, and the rate in the present study was lower than that in other sequences. Although the false positive nodules were <6 mm in diameter, they would likely not affect further treatment. The percentage of false positives depends on several factors, including scanning parameters, the CT analysis method, and nodule characteristics (40). The recommended method for reducing the false positive rate is to combine two or three sequences (33). Other recommended sequence including T2-STIR and T1-VIBE. Koyama et al. (41) had demonstrated that T2-STIR sequence was superior to T2 in term of nodule detection and lesion contrast because STIR technique make it possible for some T1-decay to be submitted for T2-decay, the contrast of tissues was higher in STIR sequence. The results of Cieszanowski et al. (42) showed that the nodules detection rate of T1-VIBE was 69%, and T1-VIBE could detect more calcified nodules than other sequences.

In the present study, 42/256 pulmonary nodules were not detected by MRI, comprising 17 SNs, 5 PSNs, and 20 GGNs, and the CT values of undetected GGNs were all below −400 HU. Among these undetected nodules, 38 were <6 mm in diameter, and the other eight are conjectured to have been missed due to respiratory motion artifacts or being adjacent to vessels. Most of the undetected nodules were located in the lower lobe and center of the lung parenchyma, an area which is greatly affected by respiratory and cardiac motion artifacts. Therefore, the density, size, and location of these nodules are important factors in influencing their detection.

PETRA showed good agreement for detecting morphologic features compared to CT. MRI could distinguish different subgroup nodules, similarly to CT image. Other shape and margins characteristics which could help to differentiate malignant and benign nodules were accurately detected by MRI with substantial to almost perfect. The results of our Bland–Altman analysis revealed a good agreement between the mean diameters of pulmonary nodules when measured by CT and MRI (ICC = 0.97); a similar finding was reported by Heye et al. (33). In the current study, a >1 mm difference between the CT and MRI measurements occurred in only 16 nodules (nine GGNs and seven PSNs), and the mean diameter of these nodules was more than 10 mm. For other nodules, differences between the mean diameter results did not exceed 1 mm. These results show higher levels of interobserver and intraobserver agreement in SNs compared with PSNs and GGNs, especially for nodules >6 mm. The present study found that the mean diameters measured by the MRI were mostly smaller than those returned by the CT, particularly for PSNs and GGNs. This is potentially due to the different conditions of patient examination; the CT required the patient to hold their breath, while the MRI was completed while the patient was breathing freely. In addition, the MRI method showed certain difficulties in determining the edge of the ground glass component with lower CT values, while nodules that differed by more than 1 mm presented with diameters of >10 mm, which did not affect follow-up or treatment during the lung cancer screening. The use of MRI (PETRA) in the follow-up study can lead to a reduction of X-ray irradiation. Therefore, MRI, particularly the PETRA sequence, is recommended as an alternative method for pulmonary nodule follow-up that can potentially be used for nodule detection and nodule type assessment.

There were a number of limitations in the present study. First, underlying lung diseases, including emphysema and pulmonary fibrosis, can affect the detection of nodules, but the present study did not assess the lung conditions of the enrolled patients. Second, the study group was comprised of only 75 patients. A larger population is required to confirm these results. Third, the study sample contained three calcified nodules, and the MRI method had limitations in identifying the calcification. Hence, there is a need to combine these results with other sequences for differentiation. Finally, due to the study restrictions, only one CT professional participated in this research; more professionals should be consulted in future studies.

## Conclusion

MRI has a potential role in detecting and evaluating pulmonary nodules, demonstrating a high level of sensitivity and a low false positive rate. The PETRA sequence has high sensitivity, particularly for nodules with diameters of >8 mm and GGNs. Furthermore, PETRA shows good agreement with CT scanning in assessing nodule size. Therefore, MRI with PETRA is considered a useful technique for lung nodule detection and may be an effective alternative approach for follow-up regarding pulmonary nodules in lung cancer screening.

## Data Availability Statement

The original contributions presented in the study are included in the article/supplementary material. Further inquiries can be directed to the corresponding author.

## Ethics Statement

The studies involving human participants were reviewed and approved by The Fourth Hospital of Hebei Medical University. The patients/participants provided their written informed consent to participate in this study.

## Author Contributions

HF and GS conceived the idea and conceptualized the study. HF, HL and YD collected the data. NZ and YW analyzed the data. HF drafted the manuscript, then GS reviewed the manuscript. All authors contributed to the article and approved the submitted version.

## Conflict of Interest

The authors declare that the research was conducted in the absence of any commercial or financial relationships that could be construed as a potential conflict of interest.
